# The Effect of Brilliant Blue-Based Plaque-Staining Agents on Aesthetic Orthodontic Appliances

**DOI:** 10.3390/ma14227050

**Published:** 2021-11-20

**Authors:** Justyna Topolska, Sylwia Motyl, Aleksandra Orłowska, Andrzej Borkowski, Paweł Działak, Krzysztof Gronkiewicz

**Affiliations:** 1 Faculty of Geology, Geophysics and Environmental Protection, AGH University of Science and Technology, 30-059 Kraków, Poland; aborkowski@agh.edu.pl (A.B.); dzialak@agh.edu.pl (P.D.); 2 Department of Dental Prosthetics and Orthodontics, Dental Institute, Jagiellonian University Medical College, 31-008 Kraków, Poland; sylwia.motyl@uj.edu.pl (S.M.); krzysztof.gronkiewicz@uj.edu.pl (K.G.); 3 Orthodontics Clinic, University Dental Clinic in Krakow, 31-155 Kraków, Poland; ola.orlowska@poczta.Onet.pl

**Keywords:** orthodontic aesthetic brackets, plaque-staining fluids, colour parameters, colour-changing adhesive, oral hygiene, Brilliant Blue sorption, monocrystalline brackets, light-cured adhesive materials

## Abstract

Orthodontic appliances discolour over treatment time, and a yellowish plaque builds up on the contact area of the brackets, adhesive and teeth. Brilliant Blue-based plaque-staining agents (BBPSAs), which increase tooth brushing efficiency, have the potential to support the maintenance of proper oral hygiene during orthodontic treatment. However, they exhibit strong colouring properties, and their impact on the aesthetics of braces remains unclear. Therefore, the aim of this study was to investigate the influence of commercially available BBPSAs on the colour of aesthetic orthodontic materials. A light-cured, colour-changing orthodontic adhesive and new-generation, monocrystalline, sapphire brackets were chosen for the experiments. The effect of the staining agent on the tested materials was investigated in terms of the reaction temperature and time, as well as the presence of black tea-induced impurities on the materials. The CIELAB (Commission Internationale de L’éclairage L* a* b*) colour system parameters were measured, and the colour differences (ΔE*ab and ΔE_00_—the Commission Internationale de L’éclairage 2000 colour-difference) were determined for the materials under several experimental conditions. The braces’ green-red colour expression was positively affected by the BBPSA. Under in vitro conditions, the regular use of the BBPSA for 90 days visibly improved the unfavourable colour change caused by the black tea.

## 1. Introduction

Improving the beauty and harmony of the face is the main motivation of patients seeking orthodontic treatment [1], and this definitely dominates among other reasons, such as elimination of malocclusion and maintenance of the long-term health of the teeth and periodontium [2]. However, a fixed orthodontic appliance—placed in the oral cavity—deteriorates the aesthetic perception of the face during orthodontic treatment. Therefore, to meet the aesthetic needs of patients, plastic and ceramic materials have been used instead of metal alloys, introducing a transparent, edgewise bracket system (the so-called “aesthetic”). The early plastic brackets were made of polycarbonate, and their main disadvantage was easy discolouration and deformation [1]. To improve the physical and mechanical properties of aesthetic brackets, sapphire-like or zirconium mono- or poly-crystals have been used in production [2,3,4]. Moreover, for the most demanding patients, a customised aesthetic ceramic bracket system produced using 3D printing technology has recently been proposed [5,6]. The manufacturing process strongly affects the properties of braces [7,8,9,10]. In general, the monocrystalline brackets included in the translucent brackets group are found to be more aesthetic than the non-translucent, polycrystalline parts of the appliance [11,12]. The adhesive material that binds the brackets to the enamel surface is an indispensable component, complementing the fixed orthodontic appliance and strongly affecting its aesthetic perception. Adhesive pastes are applied manually by the operator and cured during the polymerisation process. The polymerisation course is one of the factors determining the aesthetics during bracing and treatment. Hence, the choice of bonding material is crucial in the case of patients with high aesthetic requirements [13]. Light-cured materials are especially acknowledged by orthodontists, as they enable precise positioning of the bracket on the tooth crown and cleaning of the excess material around the bracket base [14]. To simplify the removal of excess material, some orthodontic adhesives are initially coloured and become transparent after polymerisation [15]. Thus, the use of light-cured, colour-changing orthodontic adhesives in combination with new-generation, monocrystalline brackets has become the standard in treatment with aesthetic orthodontic appliances.

However, even the highest quality brackets and adhesives of the aesthetic system wear out and discolour during orthodontic treatment [16]. These colour alterations are irreversible, as they are derived from the properties of the material and its surface, such as superficial roughness or shape [17,18,19]. However, in the case of adhesive materials, light-induced or chemically induced polymerisation may also influence their further discolouration [20,21]. Active orthodontic treatment lasts from several months to several (two or three) years. During this time, the aesthetics of the orthodontic materials change through the adsorption of dyes and plaque. The average human diet is rich in substances such as black tea, coffee, yoghurt with fruits, cherry juice, curry, cola and red wine, which contain strong pigments [22]. These staining substances cause enamel discolouration and affect the resin-based adhesive composites and orthodontic braces [11].

Unlike the discolouration caused by staining substances, the accumulation of dental plaque by orthodontic appliances is not just an aesthetic problem [23]. Plaque strongly increases the risk of caries and gingivitis, which, during orthodontic tooth movement, may additionally aggravate inflammation in the periodontium, resulting from tooth displacement [24,25,26]. Therefore, orthodontic patients should pay special attention to the care of oral hygiene, and it should be mentioned that they face a real challenge in this regard. On the one hand, regular and thorough tooth brushing along with the use of effective hygienic measures prevents caries, plaque formation, white spots on the enamel and gingivitis [27,28,29]. On the other hand, an improper and too intense cleaning procedure may cause the accidental deboning of brackets, which causes further enamel microcracks, scratches and abrasions [28]. There are many chemicals on the market that support the daily care of proper oral hygiene. Some of them tint the plaque, which allows for its precise and gentle removal by brushing. In this regard, products based on the food colouring CI 42090 Brilliant Blue are particularly popular. They are commercially available and recommended for children, as they appear fun, which improves tooth brushing. These products could be extremely helpful for orthodontic patients to maintain proper oral hygiene, which is crucial during treatment. Furthermore, it is worth mentioning that, unlike the yellow shade of dental plaque or the brown coating from beverages, a slight light blue tint of the appliance on the teeth might be perceived as an overall positive aesthetic effect, giving the impression of whiter teeth [30].

However, to date, no studies investigating the effect of Brilliant Blue-based plaque-staining agents (BBPSAs) on the aesthetic components of orthodontic appliances have been reported. Moreover, some manufacturers of BBPSAs recommend their use during treatment with fixed orthodontic appliances, while others warn that the contained food dye may stain parts of the braces placed on the teeth. To date, there are no reliable data on the possible implications of using BBPSAs for the optical parameters of aesthetic appliances. Furthermore, the course of the potential colouring reaction and its intensity have not yet been described. Therefore, the potential of using Brilliant Blue-based colouring agents to support oral hygiene during orthodontic treatment remains unexplored. Hence, the objective of the present study was to investigate the effect of a Brilliant Blue-based plaque-staining agent on the colour parameters of an aesthetic orthodontic appliance. The effect of the staining agent on the tested materials was investigated in terms of the reaction temperature and time, as well as the presence of black tea-induced impurities on the materials. The experiments were also supported by an analysis of the BBPSA’s sorption on the adhesive material. The production of orthodontic appliances is a non-standardised process in terms of the materials used, shapes of the manufactured elements and their surface finish. There are no proper reference materials allowing for scientific research that is representative for all materials available on the market [11]. Therefore, in this study, a set of brackets and adhesive materials well recognised by specialists was selected for testing. A light-cured, colour-changing orthodontic adhesive and new-generation, monocrystalline, sapphire brackets were chosen for the experiments. The materials are considered as state of the art in terms of providing high aesthetics during treatment. We believe that, in this respect, the selected experimental setup can be used as reference material for subsequent tests and other studies.

## 2. Materials and Methods

### 2.1. Orthodontic Appliances Components

A set of state-of-the-art, commercially available materials comprising the aesthetic brackets and colour-changing adhesive material was tested in this study (Table 1). The M1 sample code was used only for the brackets. The Radiance Plus brackets (American Orthodontics, Sheboygan, WI, USA) were used, as they are considered as one of the clearest, commercially available brackets [12]. The manufacturer states that the single crystal from which the brackets are made is honed and heat polished. This process is supposed to ensure a thorough smoothing of microcracks and other flaws that may be a reservoir for dyes. The applied Quad Matte^TM^ base is to provide a strong retention in the central part of the bracket and is delicate on the circuit. Due to this, the bracket should firmly bond to the enamel surface and be easily and predictably debonded. In all experiments in this study, the brackets for the right, upper incisor were used. The M2 sample code was used for the brackets with the adhesive material. Transbond™ PLUS Colour Change Adhesive (3M Unitek, St. Paul, MN, USA) was used in the experiments and applied to the brackets according to the following procedure [26]:

1. Apply the Transbond™ PLUS Colour Change Adhesive (3M Unitek Orthodontic Products, Monrovia, CA, USA) to the base of the brackets held in orthodontic tweezers;
2. Press the adhesive to the bracket base (adhesive layer on the bracket base <0.2 mm);
3. Remove the excess adhesive along the edges of the bracket with a dental probe;
4. Cure the adhesive with an Ortholux™ Luminous Curing Light polymerisation lamp (1600 mW cm^−2^) (3M Unitek Orthodontic Products, Monrovia, CA, USA) for 3 s through the full thickness of the bracket.

For each material tested, the total number of 45 samples was analysed, which means that 90 brackets and 45 portions of the adhesive material were used in this study. Additionally, the adhesive material was addressed in the sorption experiments in the amount of 10 capsules (2 mm thick and 5 mm in diameter).

### 2.2. Immersion Solutions

Two types of immersion solutions with the assumed opposite effect on the orthodontic appliances were used for this study: a Brilliant Blue-based plaque-staining agent and black tea. Both fluids are commercially available. The black tea was selected for the research as it is consumed daily by people around the world, including children [22]. The Lipton tea used in this study is a product available worldwide and is therefore often used for scientific research [31]. In turn, the plaque-colouring fluid (also referred to in this manuscript as a hygienic fluid or a plaque-staining agent) was selected based on four composition criteria: (i) it contained food dye CI 42090 Brilliant Blue; (ii) the method of dosing the liquid allowed for sorption tests of the dye in a wide range of concentrations (please refer to Section 2.3); (iii) the ingredients were specified by the manufacturer on the packaging; (iv) it contained a relatively small number of other ingredients. A detailed summary of the immersion solution parameters is presented in Table 2.

### 2.3. Experiements

Please note that an overview of the experimental setup is summarised in Appendix A Table A1.

#### 2.3.1. The Effect of the Plaque-Staining Agent on the Aesthetic Orthodontic Appliance: Time-Dependent Experiment

In this experiment, the specimens of the M1 and M2 materials (Table 1) described in Section 2.1 were immersed in the plaque-staining agent (prepared as described in Table 2 for 15 s, 45 min or 9 h). The experimental times corresponded to (i) the one-time immersion recommended by the manufacturer; (ii) the total contact time of the orthodontic appliance with the fluid during its twice-daily, 3-month use; and (iii) the total contact time of the orthodontic appliance with the fluid during its twice-daily, 3-year use. The reactions were performed in sterile, disposable, 50 mL Falcon tubes, and the reactors were rotary shaken at a speed of 80 rpm at room temperature (in a GFL 3033 incubator, Gesellschaft für Labortechnik, Burgwedel, Germany). The amount of the immersion solutions was 15 mL/specimen. The experiment was repeated three times, and then the samples were subjected to a colour analysis (please refer to Section 2.4). A new set of samples was used for each time point and in each repetition. The samples without contact with the solutions were used as the controls.

#### 2.3.2. The Effect of the Plaque-Staining Agent on the Aesthetic Orthodontic Appliance: Temperature-Dependent Experiment

In this experiment, the specimens of the M1 and M2 materials (Table 1) described in Section 2.1 were immersed in the plaque-staining agent (prepared as described in Table 2) at 5 °C and 50 °C for 12 h. The fluid is adapted so that it can be diluted by the user; therefore, the temperatures selected in this experiment included extreme values from the temperature range that could be used in a home procedure (e.g., water from the refrigerator or boiled and slightly cooled water). Since only minor changes in the colour of the tested materials were expected as a result of the immersion in the BBPSA, a long reaction time of 12 h was selected. This significantly exceeded the total time of the potential exposure of the fixed orthodontic appliance to the hygiene fluids during treatment. The reactions were performed in sterile, disposable, sealed 50 mL Falcon tubes, and the reactors were shaken at a speed of 80 rpm (in a Labnet Rocker 25 gyratory shaker (Labnet International Inc., Edison, NJ, USA) combined with a Whirlpool refrigerator or a ThermoScientific Heratherm incubator (Thermo Fisher Scientific Inc., Waltham, MA, USA). The amount of the immersion solutions was 15 mL/specimen. The experiment was repeated three times, and then the samples were subjected to a colour analysis (please refer to Section 2.4). A new set of samples was used for each temperature and in each repetition.

#### 2.3.3. The Effect of the Plaque-Staining Agent on the Tea-Stained Aesthetic Orthodontic Appliance

The experiment was designed to simulate, in a simplified way, the daily contact of the fixed orthodontic appliance with the selected staining fluids. Each experimental setup consisted of 3 specimens of the M1 material and of 3 specimens of the M2 material exposed to the immersion cycles as presented in Table 3. Three groups of the experimental setups were used, for which the experiment was terminated after 30, 60 or 90 cycles. The experiment was repeated three times, and then the samples were subjected to a colour analysis (please refer to Section 2.4).

The two orthodontic materials (M1 and M2) described in Section 2.1 were tested in the experiment. The immersion solutions were prepared fresh before each cycle as described in Section 2.2 and presented in Table 2. The amount of the immersion solutions was 15 mL/specimen. The reactions were performed in disposable, sterile, 50 mL Falcon tubes, and the reactors were rotary shaken at a speed of 80 rpm at 37 °C (in a GFL 3033 incubator). A new set of samples was used for each time point and in each repetition. The samples without contact with the solutions were used as the starting time point.

#### 2.3.4. Sorption Experiment

Sorption experiments of the plaque-staining agent on the adhesive material were performed. A total of 10 capsules of the Transbond™ PLUS Colour Change adhesive (2 mm thick and 5 mm in diameter) were polymerised as described in Section 2.1, then homogenised in a mortar and air dried. The adsorption process was realised as follows: the previously prepared adhesive samples, weighing 0.025 g each, were placed in glass vessels (10 mL). Then, 1 mL of the solution of the investigated compound in an initial concentration range of 0.001–0.10 mg mL^−1^ for Brilliant Blue FCF (CAS: 3844-45-9) was added. In the case of the plaque-staining agent’s solution, the spectroscopic equivalent to the Brilliant Blue solutions (measured as absorbance at 620 nm) was used due to the complex composition of the hygiene fluid. The vessels were sealed, and the mixtures were stirred for 4 h. Next, the mixtures were centrifuged at 6000× *g* for 2 min. The concentration of the Brilliant Blue or the plaque-staining agent (as equivalent to the Brilliant Blue solutions) in the supernatants (equilibrium concentration *C_eq_*) was measured using VarioskanLux (ThermoScientific, Life Technologies Holdings Pte Ltd., Singapore 739256, Singapore). The concentrations of the stains in the solutions before the sorption (initial concentration *C*_0_) were also measured. The experiments were conducted at a temperature of 25 °C. The analysis was performed five times, while each experiment was repeated three times. The amount of the adsorbed substance (*n*) was determined from the difference between the initial and final concentrations (under equilibrium conditions), defined as

(1) $$n=\frac{(C_{0}-C_{eq})\cdot V}{w}$$

where *n* is the amount of adsorbed compound (mg g^−1^), *C*_0_ is the initial concentration (mg L^−1^), *C_eq_* is the final (equilibrium) concentration (mg L^−1^), *V* is the volume of the solution in which the adsorption process was conducted (L), and *w* is the weight of the sorbent used as an adsorbent (g).

The Langmuir and Freundlich models of adsorption were applied. The Langmuir isotherm is described by the following equation:

(2) $$n=\frac{A_{m}\cdot K_{L}\cdot C_{eq}}{1+K_{L}\cdot C_{eq}}$$

where *n* is the amount of adsorbed compound (mg g^−1^), *A_m_* is the maximum amount of adsorbate that can form a monomolecular layer according to the Langmuir model (mg g^−1^), *K_L_* is the Langmuir constant, and *C_eq_* is the equilibrium concentration of the adsorbate (mg g^−1^).

The Freundlich isotherm is described by the equation

(3) $$n=K_{F}\cdot C_{eq}{}^{b}$$

where *K_F_* is the constant of the isotherm, *b* is a parameter of the Freundlich isotherm, and *n* and *C_eq_* have the same meanings as in the Langmuir isotherm. To fit the experimental data to the Langmuir model, a statistical spreadsheet (STATISTICA 13, StatSoft Polska, Kraków, Poland) was applied using the method of least squares for nonlinear models, while the model was fitted to the experimental data using a linearised isotherm:

(4) $$\log n=b\cdot \log C_{eq}+\log K_{F}$$

The equilibrium reaction constant *K_ads_* was characterised as a value of a derived function of the Langmuir isotherm at the equilibrium concentration *C_eq_* = 0. *K_ads_*, which was calculated in the manner described, is actually a distribution constant for the equilibrium of adsorption–desorption of adsorbate at a given temperature.

### 2.4. Colour Analysis

The M1 and M2 specimens that were subjected to the experiments detailed in Section 2.3.1, Section 2.3.2 and Section 2.3.3 were analysed in terms of their colour parameters. To measure only the permanent discolouration, a thorough cleaning procedure was applied to the samples upon termination of the experiments. After the experiment, each specimen was brushed with a toothbrush under tap water for 3 min, then ultrasonically treated with distilled water for 3 min and dried with compressed air for 5 s. The colour measurements were performed using the *CIELAB system, which is defined as a colour space, standardised in 1976 by the CIE (Commission Internationale de L’éclairage). The parameters in this system, L*, a* and b*, uniquely describe colour in a quantitative manner. The measurements were performed by means of a reflection spectrophotometer (Konica Minolta, Tokyo, Japan, 700 d) using the standard illuminate D65 over a white standard tile. A Xenon flash lamp with a filter blocking UV light was used as the light source. The aperture size was 3 mm × 6 mm, whereas the illuminating and viewing configuration was CIE diffuse/8° geometry. Measurements were repeated ten times for each specimen.

To determine the practical significance of the colour changes caused by the tea and the plaque-staining fluid in the experiment detailed in Section 2.3.3, the experimental samples were analysed for their mutual colour differences. The results were compared with the perceptibility and acceptability threshold values determined for dental and orthodontic materials. In the CIELAB colour scale system, the ΔE*_ab_ and ΔE_00_ parameters determine the interference of the individual colour components (L*, a* and b*) and mathematically define the differences in colour between two materials. Since 2001, for materials with small differences in L*, a* and b*, it is recommended to calculate the ΔE_00_ parameter rather than the ΔE*_ab_ parameter, since the former takes into account the corrections regarding a*. However, both ΔE*_ab_ and ΔE_00_ parameters have been used to report on the discolouration processes in dental and orthodontic materials [31,32,33,34]. Therefore, the ΔE*_ab_ parameter was determined using the formula [34]

ΔE∗_ab_ = [(ΔL∗)^2^ + (Δa∗)^2^ + (Δb∗)^2^]^1/2^ (5)

where ΔL* indicates changes in value, Δa* indicates changes in the red-green parameter, and Δb* indicates changes in the yellow-blue parameter between the materials for which ΔE*_ab_ is calculated. However, the ΔE_00_ parameter was calculated based on the following Commission Internationale de L’éclairage 2000 colour-difference equation (CIEDE2000) [34].

(6) $$\Delta E_{00}=\sqrt{{\left(\frac{\Delta L^{\prime }}{k_{L}S_{L}}\right)}^{2}+{\left(\frac{\Delta C^{\prime }}{k_{C}S_{C}}\right)}^{2}+{\left(\frac{\Delta H^{\prime }}{k_{H}S_{H}}\right)}^{2}+\Delta R_{T}\left(\frac{\Delta C^{\prime }}{k_{C}S_{C}}\right)\left(\frac{\Delta H^{\prime }}{k_{H}S_{H}}\right)}$$

where Δ*C*′ and Δ*H*′ are the differences in chroma and hue for a pair of specimens. Based on literature reports, the perceptibility threshold values (50:50% PT) were determined to be ΔE*_ab_ = 1 and ΔE_00_ = 0.80, whereas the values for the acceptability threshold (50:50% AT) were ΔE*_ab_ = 2.7 and ΔE_00_ = 1.8 [33,35].

### 2.5. Statistics

The statistical software STATISTICA 13 (StatSoft Polska, Kraków, Poland) was used to analyse the obtained data. Both experimental and analytical replicates were taken into consideration. The normal distribution was verified with the use of the Kolmogorov–Smirnov (K–S) test with Lilliefors correction (K–S–L) and Shapiro–Wolf test (S–W). The normal distribution data were subjected to a two-way factorial ANOVA (Analysis of Variance) analysis along with the Tukey post hoc test. The material type (M1 and M2) and the experimental setups were used as the factors. The statistical significance parameter of *p* < 0.05 is used to indicate significant difference throughout this paper.

## 3. Results

### 3.1. The Effect of the Plaque-Staining Agent on the Aesthetic Orthodontic Appliance: Time-Dependent Experiments

**Figure 1:**
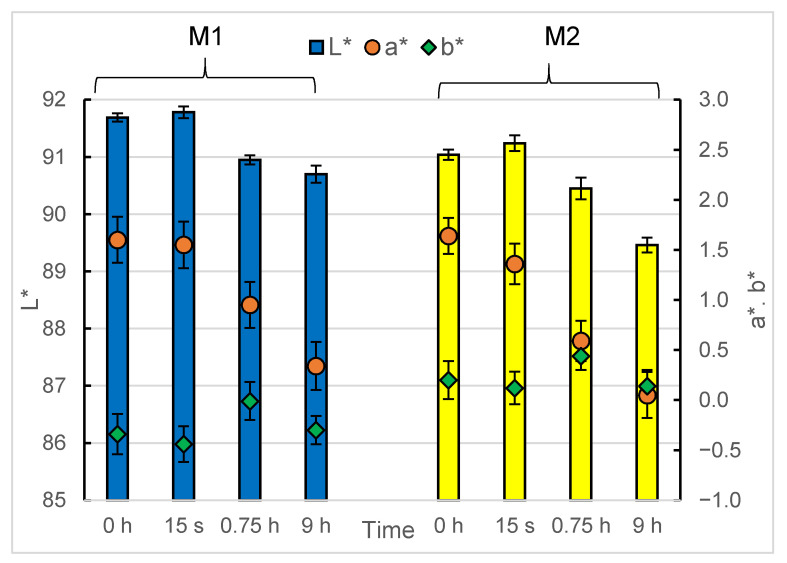
The plaque-staining agent-induced change in the experimental materials’ CIELAB parameters with the reaction time: L* (lightness) presented in the form of columns, a* (expression of the red-green wavelengths) presented in the form of filled, red circles; b* (expression of the yellow-blue wavelengths) presented in the form of filled, green diamonds. M1—the brackets, M2—the brackets with the adhesive material. Error bars express the experimental and analytical uncertainty calculated as a standard deviation of the replicates. The experiments were repeated 3 times, and the measurements were performed 10 times per sample. *p* < 0.05 was considered as the significant difference parameter.

Figure 1 shows the changes in the experimental materials’ colour after their reaction with the plaque-staining agent. The colour changes are expressed as the CIELAB (Commission Internationale de L’éclairage L* a* b*) colour scale components, where L* indicates changes in the value (lightness), a* indicates changes in the red-green parameter, and b* indicates changes in the yellow-blue parameter. Long-term and continuous immersion in the staining fluid had an impact on both materials, and the observed changes in their colour parameters varied over time. However, one-time immersion did not significantly affect the colour parameters of the tested materials. As presented in Figure 1 and confirmed by the statistical analysis, the values of the parameters L*, a* and b* did not change significantly after their single immersion in the staining agent, and the results are within the experimental error.

Long-term immersion in the plaque-staining agent had a negative impact on lightness. For both materials, the L* value decreased, and after a 9 h reaction with the staining fluid, a value of c.a. 1.5 units lower than that of the control samples was reached. Nevertheless, the values were still high and fluctuated in the range of 90.70–91.78 and 89.46–90.24 for the M1 and the M2 materials, respectively. It is worth noting that the presence of the adhesive material itself caused a decrease in the L* parameters of the brackets. The L* values measured at individual time points were on average 0.5 units lower for the M2 material than for the material M1. This was the expected result considering the non-transparency nature of the adhesive material compared to the clear brackets. The performed statistical analysis indicated significance of the observed changes both in the context of the impact of the immersion time and the differences between individual materials at a given time point.

Changes in the a* parameter over time showed a similar trend to that of lightness. This parameter’s values decreased over time for both specimens. After 9 h of the reaction, its values were c.a. 1.5 units lower than those of the controls, and a Student’s *t*-test confirmed the statistical significance of the observed changes for both tested materials. Interestingly, however, contrary to lightness, the presence of the adhesive material did not significantly reduce the brackets’ a* parameter: the values measured at particular time points did not show significant differences between the M1 and M2 materials. In the context of the a* values, the influence of the adhesive on the course of the reaction with the plaque-staining agent was not obvious. The results of the measurements after 45 min and 9 h of the reaction were identical within the experimental error for both materials; however, the mean values of the parameter, for both of these measurement points, were lower by c.a. 0.3 units for the material with adhesive. This may suggest that the presence of the adhesive was a factor accelerating the blue fluid-induced effect of reducing the red-green colours of the material.

Unlike the lightness and a* parameters, immersion in the plaque-staining agent did not cause statistically significant changes in the b* parameter corresponding to blue-yellow colours. Figure 1 shows the fluctuations in the value of this parameter over time for both tested materials, falling within the statistical experimental error. Nevertheless, similarly to the L* parameter, the presence of the adhesive itself influenced the b* parameter values of the brackets. All the results for the M2 samples were of c.a. 0.5 units greater than those of the M1 specimens, and the observed differences were statistically significant.

### 3.2. The Effect of the Plaque-Staining Agent on the Aesthetic Orthodontic Appliance: Temperature-Dependent Experiments

**Figure 2:**
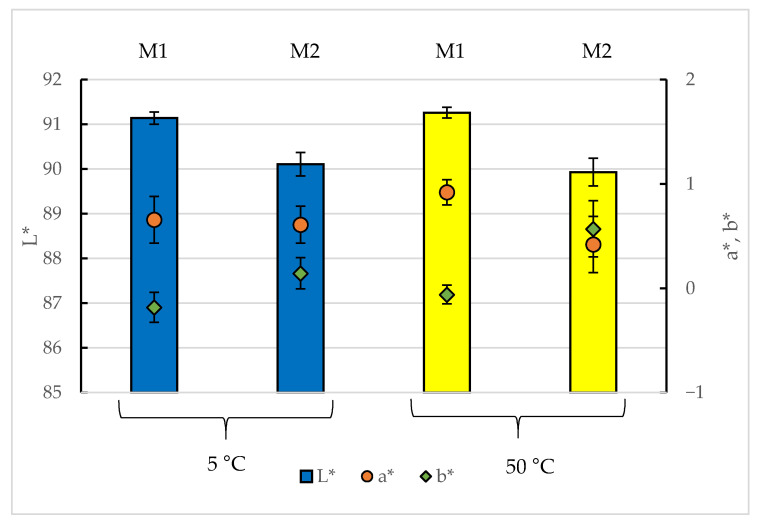
The plaque-staining agent-induced change in the experimental materials’ CIELAB parameters with the temperature of the reaction: L* (lightness) presented in the form of columns, a* (expression of the red-green wavelengths) presented in the form of filled circles; b* (expression of the yellow-blue wavelengths) presented in the form of filled diamonds. M1—the brackets; M2—the brackets with the adhesive material. Error bars express the experimental and analytical uncertainty calculated as a standard deviation of the replicates. The experiments were repeated 3 times, and the measurements were performed 10 times per sample. The *p* < 0.05 was considered as the significant difference parameter.

As presented in Figure 2, regardless of the kind of material tested, no significant impact of the temperature on the particular material’s colour parameters was observed. The obtained results of the measurements were identical within experimental error; however, some fluctuations, especially among parameters a* and b*, were observed. On the other hand, at both temperatures, the presence of the adhesive had a similar negative effect on the L* parameter. At 5 °C, the differences in the a* and b* parameters between M1 and M2 were statistically insignificant, whereas at 50 °C, the a* parameter was significantly lower, and the b* parameter was significantly higher for the material with the adhesive. This suggests that the increasing temperature affects the staining reaction of the brackets in the presence of the adhesive.

### 3.3. The Effect of the Plaque-Staining Agent on the Tea-Stained Aesthetic Orthodontic Appliance

#### 3.3.1. The Changes in the Colour Parameters over Time

**Figure 3:**
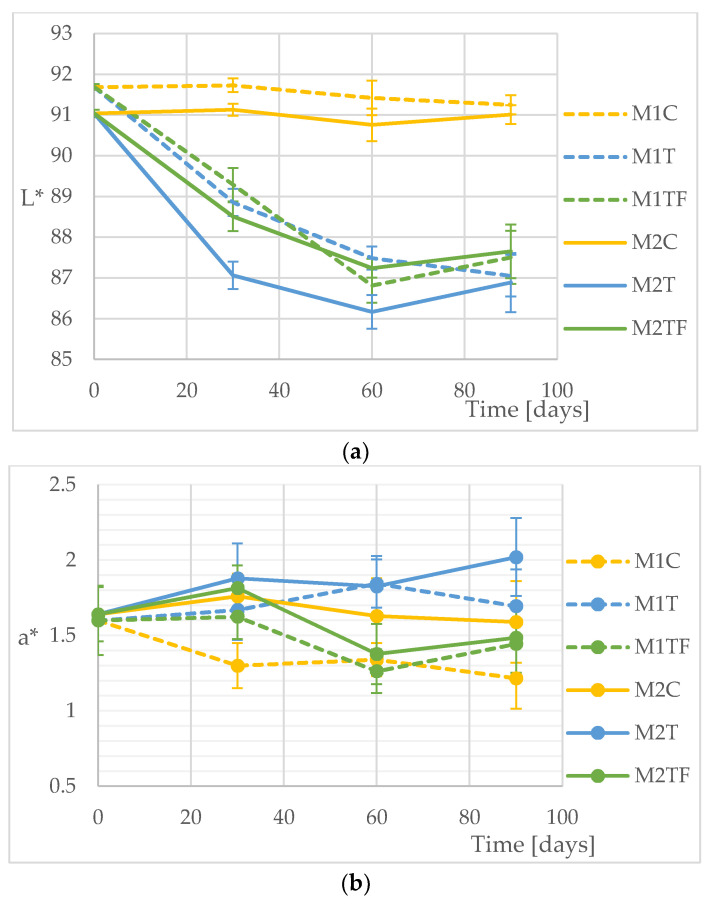

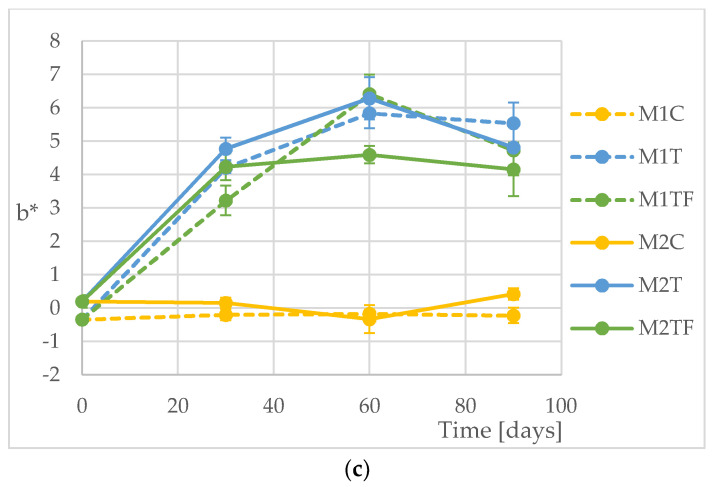
Changes in the CIELAB parameters of the experimental specimens over time: (**a**) the L* parameter (lightness); (**b**) the a* parameter (expression of the red-green wavelengths); (**c**) the b* parameter (expression of the yellow-blue wavelengths). M1—the brackets; M2—the brackets with the adhesive material; C—the control experiment; T—tea included in the reactions’ cycle; F—the plaque-staining fluid included in the reactions’ cycle. Error bars express the experimental and analytical uncertainty calculated as a standard deviation of the replicates. The experiments were repeated 3 times, and the measurements were performed 10 times per sample. *p* < 0.05 was considered as the significant difference parameter.

The black tea significantly influenced the colour parameters of the brackets. However, the results showed that the effect of the additional immersion in the plaque-staining fluid, as well as the presence of the adhesive material, was also noticeable. Figure 3a–c show the gradual changes in the colour parameters (L*, a* and b*, respectively) of the tested M1 and M2 brackets immersed in the tea solution with or without the plaque-staining agent in the reaction cycle. The measurements were made after an experimental time simulating 30, 60 and 90 days of using the hygienic fluid twice a day. Additionally, Table 4 presents the values of the L*, a* and b* parameters determined at the end of the experiment (90 simulated days), along with their statistical analysis.

As shown in Figure 3a, the presence of tea significantly affected the lightness of the materials, and at the first time point, the values for the brackets in the tea cycles were already significantly different from those of the control samples. Initially, the control samples for the M1 and M2 materials showed differences in the L* parameter, but with time, these differences decreased, and after 90 days (please refer to Table 4), they were no longer statistically significant. The L* parameters determined for the experimental samples reacting with the tea (M1T and M2T) and with additional blue-staining immersion (M1TF and M2TF) had a similar trend of changes over time. As in the case of the control samples, for these experiments, initially, the presence of adhesive had a significant effect on the measurement results, but with time, these differences became statistically insignificant (Table 4). Among all the experimental setups, the M2T material had the lowest and, thus, the worst lightness parameters: the bracket with the adhesive, reacting with tea and without the plaque-staining agent. The use of the plaque-staining agent significantly improved the lightness of the tea-stained brackets with the adhesive; at individual time points, the average values of the L* parameter for the M2TF material were about 1.5 units higher than the values for the M2T material. These differences were high enough that the results for the M2-type material treated with tea and simultaneously with the hygienic fluid (M2TF) were statistically identical to the results for the M1-type materials (brackets without adhesive). The influence of the staining agent on the course of the M1T experiment also seemed favourable in the context of the L* parameter; nevertheless, the experimental error was too large to draw unequivocal conclusions in this regard. Thus, it can be summarised that the hygienic fluid affected the lightness of the tea-stained brackets by mainly acting on the adhesive.

Figure 3b shows the variation in the expression of the red-green wavelength range with time. The figure indicates that the immersion in the plaque-staining agent affected both tea-reacting experimental materials—M1 and M2. The mean values of the a* parameter for the samples from the hygienic fluid experiments were lower than those for the samples reacting with tea only, and the observed differences increased over time. As presented in Figure 3b, the expression of the red-green colours by the materials reacting with both the plaque-staining agent and the tea tended to decrease with the experimental time, while the same materials reacting with the tea alone had an increased red-green colour expression (increasing parameter a*). It is worth mentioning that the experimental procedure itself had little effect on the samples, as for both controls, the a* parameter decreased slightly with time despite the absence of tea and the hygienic fluid in the solutions. As indicated in Table 4, at the end of the experiment, the a* parameters for both materials (M1TF and M2TF) reacting with the plaque-staining agent were statistically identical with each other and with the results of the control samples. Additionally, the M2TF material immersed in the hygienic fluid and the tea significantly differed in terms of the a* parameter from its counterpart treated with the tea alone (M2T). The fluctuations in the parameter’s value over time and the experimental error did not allow us to state similar differences for the material M1, although at the time point corresponding to 60 cycles, the differences in the values of parameter a* between materials M1T and M1TF were statistically significant. In summary, the results of the measurements of the a* parameter over time confirmed the observations for the L* parameter, indicating that the most unfavourable trend in this respect is for the brackets with the adhesive reacting with the tea alone and that the use of the plaque-staining agent had a positive effect on the expression of the red-green, tea-induced colours by both M1 and M2 materials.

Figure 3c shows the fluctuations within the b* parameter of the experimental materials over time. Reacting with tea significantly worsened the results of this parameter for all materials, ultimately distancing the experimental samples from the controls by 4–6 units. The trend of changes in the expression of the blue-yellow colours by the samples reacting with tea and the plaque-staining agent was similar for both tested materials: M1 and M2. Nevertheless, in the 60th cycle, the average b* values for the brackets with the adhesive reacting also with the hygienic fluid were the lowest among all experimental setups. The b* parameter showed considerable fluctuations over time, and although for both materials the mean values of the b* parameter were lower for the experiments with the plaque-staining agent, statistically, these results did not differ from each other. There were two deviated samples: the M1TF material at time point 30 and the M2TF material at time point 60. For both of them, their b* values were lower than those of the remaining experimental samples, taking into account the experimental error.

#### 3.3.2. The Colour Differences between Samples

To understand the total effect of the staining solutions on the tested materials, the specimens from the experiment detailed in Section 2.3.3 were analysed for their mutual colour differences. Table 5 presents the ΔE*_ab_ and ΔE_00_ parameters calculated for the individual pairs of experimental samples using Equations (5) and (6), given in Section 2.4, and the measured CIELAB parameters. The results were compared to the perceptibility (PT) and acceptability (AT) threshold values reported for dental and orthodontic materials and given in the table footer. The green and the red fonts in Table 5 indicate the results exceeding the PT and AT thresholds, respectively. Considering these threshold values, the control M1C and M2C samples were mutually identical in colour for the whole experimental time, i.e., for these samples, the calculated ΔE parameters were below the PT. However, the differences in colour between the experimental samples and the control samples ranged from ~5.00 to >8.00, significantly exceeding the AT for this type of material. Moreover, the samples treated with the tea alone (M1T and M2T) had a noticeably different colour to that of the samples reacting with the tea and the BBPSA (M1TF and M2TF). For both materials, the ΔE parameters calculated for these samples exceeded the perceptibility thresholds. The plaque-staining agent reduced the negative tea effect, as the M1TF and M2TF samples exhibited less colour change compared to the controls (M1C and M2C) than the M1T and M2T samples.

The corresponding ΔE*_ab_ and ΔE_00_ parameters calculated for the same pair of specimens differed from each other, with the value of ΔE_00_ usually being considerably lower (Table 5). This is in line with the literature reporting that for the specimens exhibiting small mutual differences in L*, a* and b*, the ΔE_00_ parameter is a more precise expression of the colour variation than ΔE*_ab_ [36,37].

### 3.4. Sorption Experiment

**Figure 4:**
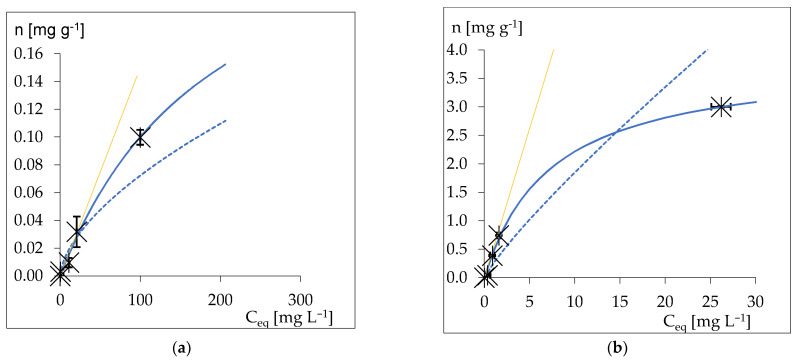
Sorption of the (**a**) plaque-staining agent (the spectroscopic equivalent to Brilliant Blue solutions) and (**b**) Brilliant Blue FCF (CAS: 3844-45-9) on the powdered adhesive material used in this study. The blue lines represent the Langmuir (solid line) and the Freundlich (dotted line) sorption models’ fit. The yellow line represents the affinity of the adsorbate to the adsorbent.

The results of the experiments detailed in Section 3.1, Section 3.2 and Section 3.3 revealed that the plaque-staining agent had a particular effect on the brackets with the adhesive. Figure 4a,b show the results of the sorption experiments for the staining agent (Figure 4a) and for the Brilliant Blue FCF (CAS: 3844-45-9) (Figure 4b) on the adhesive material used in this study. Brilliant Blue FCF (CAS: 3844-45-9) is a component of the staining agent. The parameters of the Langmuir and Freundlich sorption models are provided in the Appendix A in Table A2. As presented in Figure 4a,b, the obtained isotherms indicated the Langmuir equation as the sorption model for both solutions. This allowed an assessment of the maximum sorption capacity based on the obtained *A_m_* parameter of Equation (2). Sorption efficiency was significantly different for the two solutions tested. The sorption maxima of the staining agent and the Brilliant Blue were 0.300 mg g^−1^ and 3.84 mg g^−1^, respectively (Table A1). Similarly, the equilibrium partition constants (*K_ads_*), considered as a measure of the affinity of the dye to the adsorbent, were different for the pure Brilliant Blue dye sorption and for the sorption of Brilliant Blue as an ingredient of the staining agent. The distribution equilibrium reaction constants *K_ads_* were 0.0015 for the staining agent and 0.51 for the pure Brilliant Blue dye (Table A1). In Figure 4a,b, *K_ads_* are represented by a straight line tangent to the Langmuir function at *C_eq_* = 0. The sorption of the dye from the hygienic fluid on the adhesive material was noticeable; however, it was notably lower than that of the pure Brilliant Blue dye. This suggests that the plaque-staining agent’s other ingredients rather than the Brilliant Blue reduced the affinity of the dye to the tested adhesive material; however, they did not eliminate it completely.

## 4. Discussion

The lips are the second element of the human face determining facial beauty [36,37]. Hence, nowadays, the aesthetics of a smile is one of the most frequent reasons for undertaking orthodontic and dental treatments [38,39]. Therefore, manufacturers of dental restorative materials and aesthetic orthodontic appliances compete in offering products that not only fulfil their function during treatment but also meet the aesthetic needs of patients. The products should harmonise with the elements of the oral cavity in terms of their colour, which usually refers to their high lightness (L* parameter ~100) and neutral chroma and hue (the a* and b* parameters c.a. = 0). The materials used in the presented experiments met the highest aesthetic standards. The colour parameters of the brackets used were as follows: L* = 91.69 ± 0.07, a* = 1.6 ± 0.23 and b* = −0.34 ± 0.2. Although the application of an opaque adhesive material affected the brackets’ parameters, they remained at the values ensuring high aesthetics (L* = 91.04 ± 0.09, a* = 1.64 ± 0.18 and b* = 0.2 ± 0.19).

During treatment, the aesthetics of the best orthodontic appliances deteriorate. This is due to the adsorption of food and plaque, causing discolouration of the brackets and the adhesive materials [11,16,18,23,31,40]. The difficulty in accessing the teeth’s surface caused by the fixed appliance components may aggravate this problem [27]. Hygienic fluids that stain plaque enable its precise removal; however, there was concern that their ingredient, namely, the Brilliant Blue FCF (BBFCF) dye, would seriously affect the aesthetics of the orthodontic appliance. However, this study has revealed for the first time that Brilliant Blue-based hygienic fluid does affect the colour of aesthetic orthodontic appliances, but its influence is rather positive in this regard.

Brilliant Blue FCF is primarily known as a non-toxic dye used in the food and cosmetic industries. However, its ability to adsorb on selected surfaces has also made it applicable in medicine [41] and environmental sciences [42,43,44,45]. The characteristics of Brilliant Blue FCF sorption processes are complex. Some literature reports postulate that the sorbent’s composition and its surface properties are the dominant factors influencing the BBFCF sorption reactions [43]. Others, in turn, indicate the major role of the solution parameters in this process (such as pH, ionic strength and the presence of other substances) [44]. The results of the research carried out as part of this study confirm both above-mentioned theses.

Firstly, in both time- and temperature-dependent experiments presented herein, the effect of the BBPSA on the orthodontic brackets was enhanced in the presence of the adhesive material. This was due to the stain-resistant composition of the brackets and the difference in the quality of the surface finish of both materials [46]. The brackets used in the experiments were composed of monocrystalline alumina oxide, considered as a material with a low affinity for Brilliant Blue FCF [43]. Furthermore, the manually applied adhesive material was prone to yield a more porous and uneven surface compared to prefabricated, polished and surface-treated brackets. Hence, the surface roughness of the adhesive material was one of the factors favouring the staining processes [46].

Secondly, the time-dependent experiments indicated that the single immersion in the Brilliant Blue-based plaque-staining fluid did not cause any significant changes in the colour parameters of the tested materials, whereas the effect of the long-term immersion was, in this regard, significant. The latter was significant for both single but long-term immersion and multiple but short-term immersion. Two main factors probably determined such a result of the experiments. The first factor is related to the partly irreversible nature of the sorption process and the sorption–desorption hysteresis, which was previously reported for the Brilliant Blue FCF [45]. While a single, short-term interaction with the fluid could not confirm the observations in this regard, a long-term experiment revealed the actual course of the staining treatment. The second factor regards the composition of the plaque-staining agent, which contains surfactants likely influencing the physicochemical properties of the fluid, thus affecting the staining process. As presented in Section 3.4, the sorption maxima and the equilibrium partition constants of the BBFCF were over two orders of magnitude lower for the staining agent than for the pure BBFCF dye. The conducted sorption experiment indicated that the other ingredients of the fluid reduced the affinity of the dye to the tested material; however, they did not eliminate it completely. Therefore, only a long-term or multiple interaction of the braces with the BBPSA brought measurable and visible changes in their colour parameters. It is worth mentioning that the staining fluid changed the colour of the tested orthodontic materials mainly by reducing their a* parameter. After 9 h of immersion in the BBPSA, the expression of the green-red wavelengths (a*) decreased by 79% and 97% for the brackets and the brackets with the adhesive material, respectively. At the same time, the lightness (L*) decreased by 1% and 2%, respectively, while the value of the b* parameter (yellow-blue colour) remained within the experimental error range for both tested materials. The expression of the red colour is related to the reflection of light with a wavelength of about 620 nm. This wavelength, in turn, corresponds to the maximum light absorption by the Brilliant Blue FCF. Therefore, Brilliant Blue-based hygienic liquids have the potential to maintain the aesthetics of orthodontic brackets that are prone to browning during the course of treatment. Furthermore, it is worth mentioning that all the above-mentioned mechanisms do not affect the basic function of such fluids, which is to enable the effective removal of plaque via precise tooth brushing.

The experiments presented in Section 3.3 were designed to verify the ability of the Brilliant Blue-based plaque-staining agent to improve the aesthetics of the orthodontic appliances that deteriorated over time due to the activity of black tea. The results were interpreted regarding the colour differences that appeared between the samples undergoing different staining procedures. The interpretation of the colour differences between the two materials expressed by the ΔE parameters is based on the statistical tests determining the thresholds for the ability of the observers to notice the changes. The conventional dependency between the ΔE*_ab_ parameter and the human eye perceptibility of the difference in the colour, given by the ISO last century, is as follows:

0 < ΔE < 1—the typical observer does not notice the difference,

1 < ΔE < 2—the difference is noticed only by an experienced observer,

2 < ΔE < 3.5—the difference is also noticed by an inexperienced observer,

3.5 < ΔE < 5—the observer notices a clear difference in colours,

5 < ΔE—the observer has the impression that there are two different colours.

This scale is adjusted each time to the specifics of the industry and the materials for which the colour differences are tested, defining the perceptibility and acceptability thresholds at which 50% of observers perceive or accept the colour difference [38,39]. Due to the pioneering nature of the research presented herein, there are no reports on the thresholds for our experimental setups. Nevertheless, in the context of dental restorative materials, the reported perceptibility thresholds values range between (50:50% PT) ΔE*_ab_ = 1–1.2 and ΔE_00_ = 0.80, while the values for the acceptability thresholds range between (50:50% AT) ΔE*_ab_ = 2.7–3.7 and ΔE_00_ = 1.8 [33,35]. The lowest values from the given ranges were selected as the basis for the interpretation of the data presented in this paper (please refer to Table 5).

The results of the experiments presented in Section 3.3 indicated that the reaction with the black tea significantly deteriorated the colour parameters for both materials: the brackets and the brackets with the applied adhesive (Figure 3a–c). This was expected, as it has previously been reported in the literature [31]. However, the calculated ΔE*_ab_ and ΔE_00_ expressing the colour difference between the control samples and the tea-stained specimens exceeded the acceptance thresholds for a colour change in dental materials. Furthermore, despite the fact that the materials tested were of high aesthetic quality, after immersion in the black tea, their colour change parameter was >5. This conventionally means that they expressed colours that were completely different to those initially expressed. It is also worth mentioning that the presence of the adhesive material enhanced the effect of the black tea on the perceived colour of the brackets. For the majority of the time points, the values of the ΔE parameters calculated for the M2 specimens immersed in the tea vs. control samples were higher than those of the corresponding M1 samples (Table 5). (The effect of the surface roughness of the adhesive material on the enhanced staining process is explained above.) However, under in vitro conditions, the regular, long-term use of the BBPSA slightly improved the colour perception for both tested materials also undergoing tea staining. After 90 staining cycles, the colour changes observed for the brackets immersed in the tea and the BBPSA solutions were less intense than those of the materials only undergoing the tea cycles. The colour differences ΔE calculated for the M1T vs. M1TF and for the M2T vs. M2TF were c.a. = 1 (Table 5). This suggests that the colour improvement induced by the reaction with the BBPSA exceeded the perceptibility threshold; i.e., it was noticeable. Nevertheless, the effect was not sufficiently intense enough to reduce the tea-enhanced colour parameters below the acceptance threshold for dental materials. The colour differences ΔE calculated for the M1TF vs. M1C and for the M2TF vs. M2C were still >5 (Table 5). It is worth mentioning that the long-term immersion in the Brilliant Blue plaque-staining agent influenced the colour parameters of the tea-affected materials mainly in terms of their red-green colour expression (Table 4). As presented in Figure 3a–c, the L* and b* parameters gradually deteriorated with the staining time for all specimens except the controls. In contrast, the fluctuation of the a* parameter was of a different trend. There was a distinction between the reaction pathways for the control samples and for those also stained with the BBPSA and the samples only undergoing the tea cycle. The effect of the Brilliant Blue FCF on the expression of the green-red colour of its sorbates is due to its light absorption properties and is discussed above.

Finally, the presented results provide a solid basis for further clinical trials, as well as for the further testing of dental and orthodontic materials in terms of maintaining their aesthetics with the use of hygienic fluids during treatment. Therefore, it is worth mentioning that our experiments indicated that an elevated temperature (50 °C) may increase the effect of the plaque-staining agent on the a* and b* colour parameters of the adhesive. It was also shown that the change in the temperature in the range between 5 °C and 50 °C did not significantly influence the effect of the hygienic fluid on the brackets. This suggests that the fluid temperature factor should be considered during comparative studies on dental material discolouration.

## 5. Conclusions

Our research showed that hygienic agents containing Brilliant Blue FCF (BBPSA) can be useful for maintaining the desired level of aesthetics during orthodontic treatment. While providing the means of proper tooth brushing, BBPSAs may also be one of the factors directly reducing the negative tea-induced discolouration of aesthetic orthodontic appliances. Even after long-term immersion in a Brilliant Blue-based plaque-staining agent, the appliance exhibited the colour parameters typical of products with high aesthetic quality.

## Figures and Tables

**Table 1:** Materials used in this study.

Samples Code		Material	Brand	Batch Number	Manufacturer	Country
M2	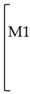	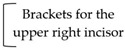	Radiance Plus with a Quad Matte^TM^ base	SOI24509	American Orthodontics	USA
		Colour-changing adhesive	Transbond™ PLUS	7120101	3M Unitek Orthodontic Products	USA

**Table 2:** Immersion solutions used in this study.

Code	Immersion Solution	Concentration	Brand/Batch Number	Manufacturer	Country
T	Tea solution	1 g/100 mL in DW * [31]	Lipton/ L1 091 1 0 620	Lipton, Unilever Polska Sp. z o.o.	Poland
F or BBPSA	Plaque-staining fluid	** 5 mL/100 mL in DW	Dropingo/ 01AF0520	ALOFARM FARMACJA POLSKA Sp. z o.o.	Poland

* Distilled water; ** in accordance with the manufacturer’s recommendations.

**Table 3:** Experimental setup: immersion cycles used.

	Cycle Step		Immersion Solutions for the M1C and M2C Samples *	Immersion Solutions for the M1T and M2T Samples *	Immersion Solutions for the M1TF and M2TF Samples *
	No.	Time			
Immersion Cycle	1.	1 min	Distilled water	Distilled water	Distilled water
	2.	15 s	Distilled water	Distilled water	Plaque-staining fluid
	3.	1 min	Distilled water	Distilled water	Distilled water
	4.	15 min	Distilled water	Tea solution	Tea solution
	5.	1 min	Distilled water	Distilled water	Distilled water
	6.	15 s	Distilled water	Distilled water	Plaque-staining fluid
	7.	1 min	Distilled water	Distilled water	Distilled water

* M1—the brackets; M2—the brackets with the adhesive material; C—the control experiment; T—tea included in the reactions’ cycle; F—the plaque-staining fluid included in the reactions’ cycle. Error bars express the experimental and analytical uncertainty calculated as a standard deviation of the replicates.

**Table 4:** Final values of the L*, a* and b* CIELAB parameters of the experimental materials.

Sample ID	CIELAB Parameters								
	L*			a*			b*		
M1C	91.25	(0.23)	a	1.22	(0.20)	a	−0.22	(0.23)	a
M1T	87.06	(0.51)	b	1.69	(0.24)	b	5.54	(0.62)	b
M1TF	87.51	(0.65)	b	1.44	(0.25)	ab	4.81	(0.75)	bc
M2TF	87.65	(0.66)	b	1.48	(0.23)	ab	4.16	(0.80)	c
M2T	86.89	(0.73)	b	2.02	(0.26)	bc	4.82	(0.66)	bc
M2C	91.01	(0.23)	a	1.59	(0.27)	abc	0.42	(0.17)	a

M1—the brackets; M2—the brackets with the adhesive material; C—the control experiment; T—tea included in the reactions’ cycle; F—the plaque-staining fluid included in the reactions’ cycle; ()—the standard deviations of the replicates’ values within a column followed by different letters differ significantly at *p* < 0.05.

**Table 5:** The colour differences between experimental specimens expressed as the ΔE*_ab_ and the ΔE_00_ parameters.

Number of Cycles	Sample ID	M1C		M1T		M1TF		M2C		M2T		M2TF	
30	M1C	0		_ϑ_ 4.54	(0.21)	_ϑ_ 5.08	(0.51)	0.63	(0.01)	_ϑ_ 5.00	(0.40)	_ϑ_ 4.36	(0.37)
60		0		_ϑ_ 5.63	(0.27)	_ϑ_ 6.26	(0.35)	0.74	(0.32)	_ϑ_ 6.36	(0.39)	_ϑ_ 4.83	(0.19)
90		0		_ϑ_ 5.45	(0.37)	_ϑ_ 4.73	(0.51)	0.56	(0.07)	_ϑ_ 5.04	(0.49)	_ϑ_ 4.27	(0.53)
30	M1T	_ϑ_ 6.09	(0.28)	0		_φ_ *0.90*	*(0.27)*	_ϑ_ 4.51	(0.23)	0.53	(0.24)	0.43	(0.20)
60		_ϑ_ 7.20	(0.35)	0		_φ_ *1.07*	*(0.20)*	_ϑ_ 5.33	(0.11)	_φ_ *0.93*	*(0.15)*	_φ_ *1.14*	*(0.17)*
90		_ϑ_ 7.14	(0.48)	0		_φ_ *0.77*	*(0.15)*	_ϑ_ 5.23	(0.43)	_φ_ *0.81*	*(0.22)*	_φ_ *1.22*	*(0.19)*
30	M1TF	_ϑ_ 5.08	(0.51)	_φ_ *1.08*	*(0.33)*	0		_ϑ_ 3.64	(0.40)	_φ_ *1.39*	*(0.13)*	_φ_ *0.89*	*(0.04)*
60		_ϑ_ 8.03	(0.45)	_φ_ *1.06*	*(0.20)*	0		_ϑ_ 6.02	(0.15)	_φ_ *0.90*	*(0.08)*	_φ_ *1.52*	*(0.31)*
90		_ϑ_ 6.21	(0.67)	_φ_ *0.96*	*(0.19)*	0		_ϑ_ 4.53	(0.59)	_φ_ *0.88*	*(0.12)*	0.50	(0.04)
30	M2C	0.63	(0.01)	_ϑ_ 5.66	(0.29)	_ϑ_ 4.66	(0.51)	0		_ϑ_ 4.95	(0.43)	_ϑ_ 4.31	(0.40)
60		0.74	(0.32)	_ϑ_ 6.98	(0.15)	_ϑ_ 7.82	(0.20)	0		_ϑ_ 6.04	(0.17)	_ϑ_ 4.53	(0.18)
90		0.78	(0.09)	_ϑ_ 6.47	(0.53)	_ϑ_ 5.56	(0.72)	0		_ϑ_ 4.77	(0.55)	_ϑ_ 4.05	(0.62)
30	M2T	_ϑ_ 6.64	(0.53)	0.63	(0.28)	_φ_ *1.68*	*(0.15)*	_ϑ_ 6.19	(0.54)	0		0.67	(0.09)
60		_ϑ_ 8.34	(0.51)	_φ_ *1.40*	*(0.23)*	0.87	(0.08)	_ϑ_ 8.06	(0.22)	0		_φ_ *1.60*	*(0.33)*
90		_ϑ_ 6.71	(0.66)	0.81	(0.22)	0.85	(0.12)	_ϑ_ 6.04	(0.70)	0		_φ_ *0.99*	*(0.14)*
30	M2TF	_ϑ_ 5.73	(0.49)	0.61	(0.29)	_φ_ *1.04*	*(0.05)*	_ϑ_ 5.28	(0.50)	_φ_ *0.94*	*(0.12)*	0	
60		_ϑ_ 6.34	(0.25)	_φ_ *1.35*	*(0.20)*	_φ_ *1.87*	*(0.38)*	_ϑ_ 6.06	(0.24)	_φ_ *2.06*	*(0.42)*	0	
90		_ϑ_ 5.67	(0.71)	_φ_ *1.52*	*(0.23)*	0.59	(0.05)	_ϑ_ 5.02	(0.76)	_φ_ *1.14*	*(0.16)*	0	

M1—the brackets; M2—the brackets with the adhesive material; C—the control experiment; T—tea included in the reactions’ cycle; F—the plaque-staining fluid included in the reactions’ cycle; ()—the standard deviations of the replicates; shaded area indicates the ΔE*ab parameters, plain area indicates the ΔE_00_ parameters; φ—the values exceeding the assumed perceptibility thresholds ΔE*ab = 1 [33], ΔE_00_ = 0.80 [35]; ϑ—the values exceeding the assumed acceptability thresholds ΔE*_ab_ = 3.7 [33], ΔE_00_ = 1.8 [35].

## Data Availability

Not applicable.

## Appendix A

**Table A1:** Summary of the overview of the experimental setups.

	Variable Tested	Material Tested	Temperature	Experimental Time	Solutions Used	Solution/Solid Ratio	Analysis
Experiment 1	Immersion time	M1 * and M2 *	Room temperature (25 °C)	15 s 45 min 9 h	BBPSA *	15 mL/specimen	Colour analysis
Experiment 2	Immersion temperature	M1 and M2	5 °C or 50 °C	12 h	BBPSA	15 mL/specimen	Colour analysis
Experiment 3	Presence of the tea impurities formed during simulated days	M1 and M2	37 °C	30, 60, 90 simulated days	Distilled water Black tea BBPSA	15 mL/specimen	Colour analysis
Experiment 4	Sorption capability toward BBPSA or Brilliant Blue FCF	Powdered adhesive material	Room Temperature (25 °C)	4 h	BBPSA Brilliant Blue FCF	1 mL/0.025 g	Brilliant Blue FCF Concentration prior and after sorption experiment

* M1—the brackets; M2, * M2—the brackets with the adhesive material, * BBPSA—Brilliant Blue-based plaque-staining agent.

**Table A2:** Parameters of the Langmuir and Freundlich adsorption model for the staining agent and the Brilliant Blue dye on powdered adhesive material.

	T	Langmuir Model			Freundlich Model			*K_ads_*
		*A_m_*	*K_L_*	*r*	*K_F_*	*b*	*r*	
Staining agent	298 K	0.30	0.005	0.99	0.0046	1.67	0.95	0.0015
Brilliant Blue	298 K	3.84	0.136	0.99	0.2675	1.17	0.90	0.5224

T—temperature (K); *A_m_*—maximum amount of adsorbate (mg g^−1^); *K_L_*—Langmuir constant; r—correlation coefficient; *K_F_*—constant of the isotherm, *b*—parameter of the Freundlich isotherm; *K_ads_*—equilibrium partition constant.

## References

[1] Alkire R.G., Bagby M.D., Gladwin M.A., Kim H. (1997). Torsional Creep of Polycarbonate Orthodontic Brackets. Dent. Mater..

[2] Kusy R.P. (2002). Orthodontic Biomaterials: From the Past to the Present. Angle Orthod..

[3] Swartz M.L. (1988). Ceramic Brackets. J. Clin. Orthod..

[4] Russell J.S. (2005). Current Products and Practice. J. Orthod..

[5] Yang L., Yin G., Liao X., Yin X., Ye N. (2019). A Novel Customized Ceramic Bracket for Esthetic Orthodontics: In Vitro Study. Prog. Orthod..

[6] Tartaglia G., Mapelli A., Maspero C., Santaniello T., Serafin M., Farronato M., Caprioglio A. (2021). Direct 3D Printing of Clear Orthodontic Aligners: Current State and Future Possibilities. Materials.

[7] Liu J.-K., Chung C.-H., Chang C.-Y., Shieh D.-B. (2005). Bond Strength and Debonding Characteristics of a New Ceramic Bracket. Am. J. Orthod. Dentofac. Orthop..

[8] Omana H.M., Moore R.N., Bagby M.D. (1992). Frictional Properties of Metal and Ceramic Brackets. J. Clin. Orthod..

[9] Ghafari J. (1992). Problems Associated with Ceramic Brackets Suggest Limiting Use to Selected Teeth. Angle Orthod..

[10] Elekdag-Türk S. (2020). In Vitro Evaluation of a Ceramic Bracket with a Laser-Structured Base. BMC Oral Health.

[11] de Oliveira C.B., Maia L.G.M., Santos-Pinto A., Júnior L.G.G. (2014). In Vitro Study of Color Stability of Polycrystalline and Monocrystalline Ceramic Brackets. Dent. Press J. Orthod..

[12] Filho H.L., Maia L.E., Araújo M.V.A., Ruellas A.C.O. (2012). Influence of Optical Properties of Esthetic Brackets (Color, Translucence, and Fluorescence) on Visual Perception. Am. J. Orthod. Dentofac. Orthop..

[13] Gange P. (2015). The Evolution of Bonding in Orthodontics. Am. J. Orthod. Dentofac. Orthop..

[14] Yılmaz B., Bakkal M., Kurt B.Z. (2020). Structural and Mechanical Analysis of Three Orthodontic Adhesive Composites Cured with Different Light Units. J. Appl. Biomater. Funct. Mater..

[15] Türkkahraman H., Adanir N., Gungor A.Y., Alkis H. (2010). In Vitro Evaluation of Shear Bond Strengths of Colour Change Adhesives. Eur. J. Orthod..

[16] Çörekçi B., Irgın C., Malkoç S., Ozturk B., Irgin C. (2010). Effects of Staining Solutions on the Discoloration of Orthodontic Adhesives: An In-Vitro Study. Am. J. Orthod. Dentofac. Orthop..

[17] Khokhar Z.A., Razzoog M.E., Yaman P. (1991). Colour Stability of Restorative Resins. Quintessence Int. Berl. Ger..

[18] Faltermeier A., Behr M., Müßig D. (2007). In Vitro Colour Stability of Aesthetic Brackets. Eur. J. Orthod..

[19] Iazzetti G., Burgess J.O., Gardiner D., Ripps A. (2000). Colour Stability of Fluoride-Containing Restorative Materials. Oper. Dent..

[20] Dietschi D., Campanile G., Holz J., Meyer J.-M. (1994). Comparison of the Color Stability of Ten New-Generation Composites: An In Vitro Study. Dent. Mater..

[21] Leibrock A., Rosentritt M., Lang R., Behr M., Handel G. (1997). Colour Stability of Visible Light-Curing Hybrid Composites. Eur. J. Prosthodont. Restor. Dent..

[22] Neves M.F., Trombin V.G., Lopes F.F., Kalaki R., Milan P. (2011). World Consumption of Beverages. The Orange Juice Business.

[23] De Freitas A.O.A., Marquezan M., Nojima M.D.C.G., Alviano D.S., Maia L.C. (2014). The Influence of Orthodontic Fixed Appliances on the Oral Microbiota: A Systematic Review. Dent. Press J. Orthod..

[24] Kumar P.S. (2013). Oral Microbiota and Systemic Disease. Anaerobe.

[25] Yamaguchi M., Fukasawa S. (2021). Is Inflammation a Friend or Foe for Orthodontic Treatment? Inflammation in Orthodontically Induced Inflammatory Root Resorption and Accelerating Tooth Movement. Int. J. Mol. Sci..

[26] Proffit W.R., Jr H.W.F., Sarver D.M. (2006). Contemporary Orthodontics.

[27] Cantekin K., Celikoglu M., Karadas M., Yildirim H., Erdem A. (2011). Effects of orthodontic Treatment with Fixed Appliances on Oral Health Status: A Comprehensive Study. J. Dent. Sci..

[28] Sandison R.M. (1981). Tooth Surface Appearance after Debonding. Br. J. Orthod..

[29] Øgaard B., Rølla G., Arends J., Cate J.T. (1988). Orthodontic Appliances and Enamel Demineralization Part 2. Prevention and Treatment of Lesions. Am. J. Orthod. Dentofac. Orthop..

[30] Sikri V.K. (2010). Color: Implications in Dentistry. J. Conserv. Dent..

[31] Lee Y.-K., Powers J.M. (2007). Combined Effect of Staining Substances on the Discoloration of Esthetic Class V Dental Restorative Materials. J. Mater. Sci. Mater. Electron..

[32] Kim H. (2020). Optical and Mechanical Properties of Highly Translucent Dental Zirconia. Materials.

[33] Khashayar G., Bain P.A., Salari S., Dozic A., Kleverlaan C.J., Feilzer A.J. (2014). Perceptibility and Acceptability Thresholds for Colour Differences in Dentistry. J. Dent..

[34] Luo M.R., Cui G., Rigg B. (2001). The Development of the CIE 2000 Colour-Difference Formula: CIEDE2000. Color Res. Appl..

[35] Paravina R.D., Ghinea R., Herrera L.J., Della Bona A., Igiel C., Linninger M., Sakai M., Takahashi H., Tashkandi E., Perez M.D.M. (2015). Color Difference Thresholds in Dentistry. J. Esthet. Restor. Dent..

[36] Goldstein R.E. (1969). Study of Need for Esthetics in Dentistry. J. Prosthet. Dent..

[37] Batwa W. (2018). The Influence of the Smile on the Perceived Facial Type Esthetics. BioMed Res. Int..

[38] Jawad Z., Bates C., Hodge T. (2015). Who Needs Orthodontic Treatment? Who Gets It? And Who Wants It?. Br. Dent. J..

[39] Armalaite J., Jarutiene M., Vasiliauskas A., Sidlauskas A., Svalkauskiene V., Skarbalius G., Sidlauskas M. (2018). Smile Aesthetics as Perceived by Dental Students: A Cross-Sectional Study. BMC Oral Health.

[40] Da Silva A.V.M., De Mattos G.V., Kato C.M., Normando D. (2012). In Vivo Color Changes of Esthetic Orthodontic Ligatures. Dent. Press J. Orthod..

[41] Hocking K.M., Luo W., Li F.D., Komalavilas P., Brophy C., Cheung-Flynn J. (2016). Brilliant blue FCF is a Nontoxic Dye for Saphenous Vein Graft Marking that Abrogates Response to Injury. J. Vasc. Surg..

[42] Nobles M., Wilding L., Lin H. (2010). Flow Pathways of Bromide and Brilliant Blue FCF Tracers in Caliche Soils. J. Hydrol..

[43] Ketelsen H., Meyer-Windel S. (1999). Adsorption of Brilliant Blue FCF by Soils. Geoderma.

[44] Germán-Heins J., Flury M. (2000). Sorption of Brilliant Blue FCF in Soils as Affected by pH and Ionic Strength. Geoderma.

[45] Morris C., Mooney S., Young S. (2008). Sorption and Desorption Characteristics of the Dye Tracer, Brilliant Blue FCF, in Sandy and Clay Soils. Geoderma.

[46] Reis A.F., Giannini M., Lovadino J.R., Ambrosano G.M. (2003). Effects of Various Finishing Systems on the Surface Roughness and Staining Susceptibility of Packable Composite Resins. Dent. Mater..

